# Supplementary material for: Evaluating frequency and quality of pathogen-specific T cells
Source: Nat Commun. 2016 Oct 27;7:13264. doi: 10.1038/ncomms13264 (PMC5095286; doi:10.1038/ncomms13264)
Supplement: Supplementary Information — Supplementary Figures 1-7, Supplementary Tables 1-3 and Supplementary References [file ncomms13264-s1.pdf]

SUPPLEMENTARY INFORMATION

Supplementary Figures

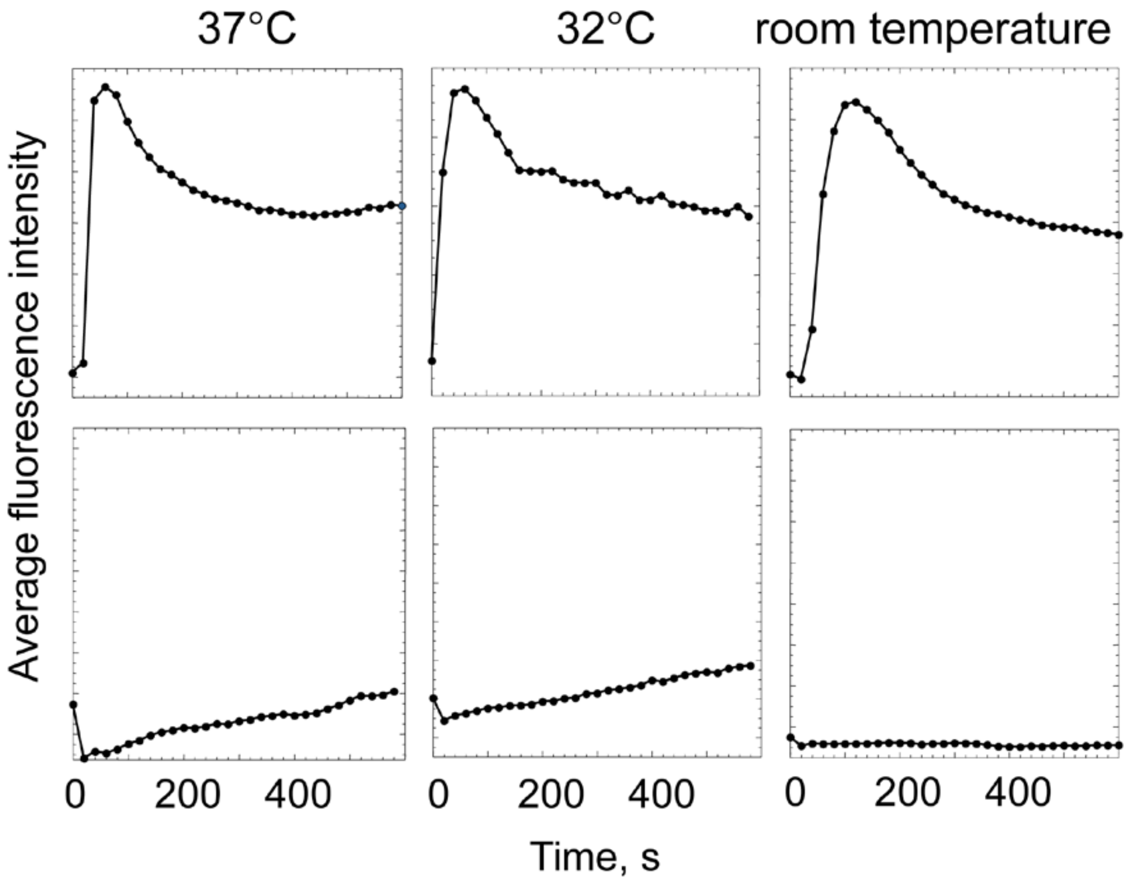

Supplementary Figure 1. Measurements of Ca^{2+} flux in the monolayer of CER43 CTL induced by cognate (upper panels) or non-stimulatory peptides (lower panels) at indicated temperatures. Cognate peptide induced intracellular Ca^{2+} flux in CER43 cells at all experimental conditions. Lowering the temperature delayed approaching maximum of the response and widened the peak width from 60 seconds to at least 120 seconds. Importantly, an optimal window of time for the signal detection did not depend on the temperature and varied within 60-120 seconds. The ratio of maximal fluorescence intensity to the background fluorescence at each temperature remained similar. Average fluorescent intensity in T cells treated with irrelevant peptide slightly increases over time at 32°C and 37°C. Analysis of these images reveals that the increased in average fluorescent intensity was due to rising of images background, but not due to increase of intracellular fluorescence. This is indicative of the fluorophore leakage from cell cytoplasm to the extracellular medium with time at a higher temperature. Consistent with this, the average fluorescent intensity of the images did not change over time at room temperature.

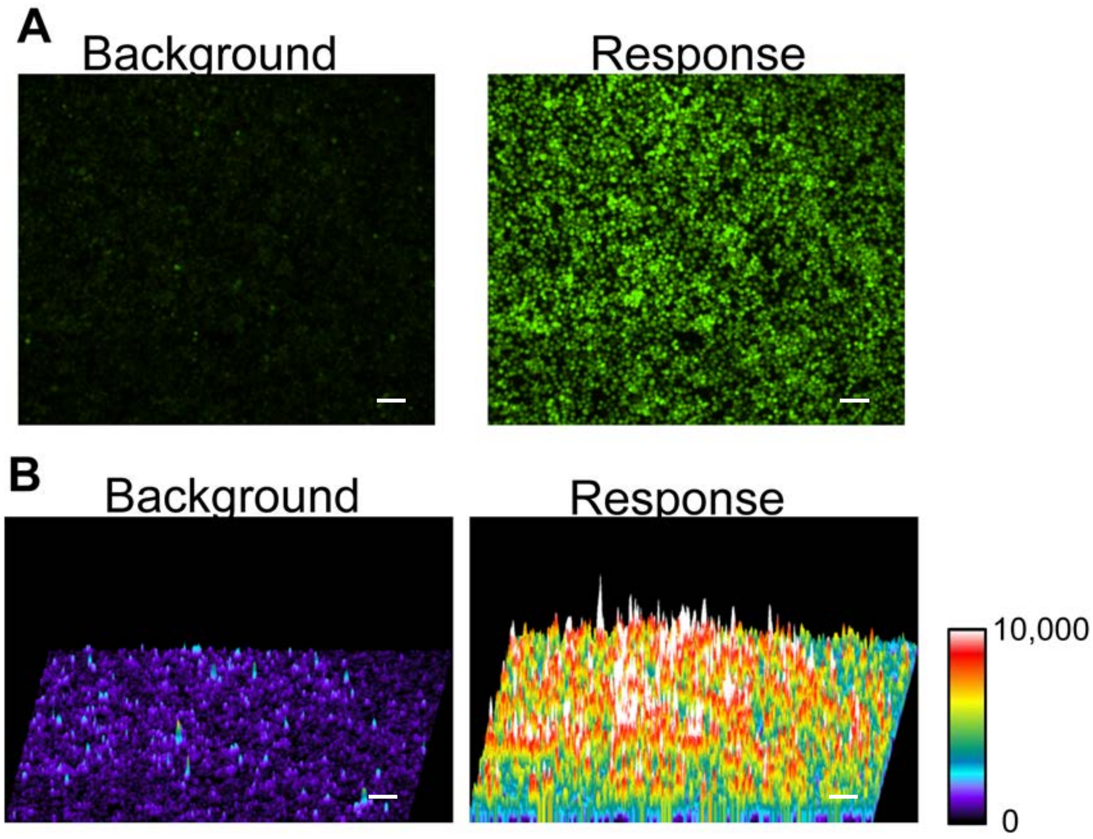

Supplementary Figure 2. Responses of CD8 CTL CER43 to strong agonist GILGFVFTL (GL9) peptide at 10^{-4} M are shown in 2D (A) and 3D (B) plots. 2D plots illustrate fluorescent intensity of CER43 cells labeled with Fluo-4 before (left) and after (right) the stimulation with GL9 peptide. 3D plots demonstrate the topographical profile of the image's fluorescent intensity level of individual cells before (left) and after (right) the stimulation. The fluorescent intensity is defined at a relative scale using pseudo colors indicated on the color bar: white and black colors correspond to maximal and minimal fluorescent intensities. The images of responding cells are taken at 220 seconds after the stimulation, i.e., at the peak of the response (see **Fig. 4**). The data shown are based on the analysis of approximately 5,000 cells. Scale bars are 50 μm.

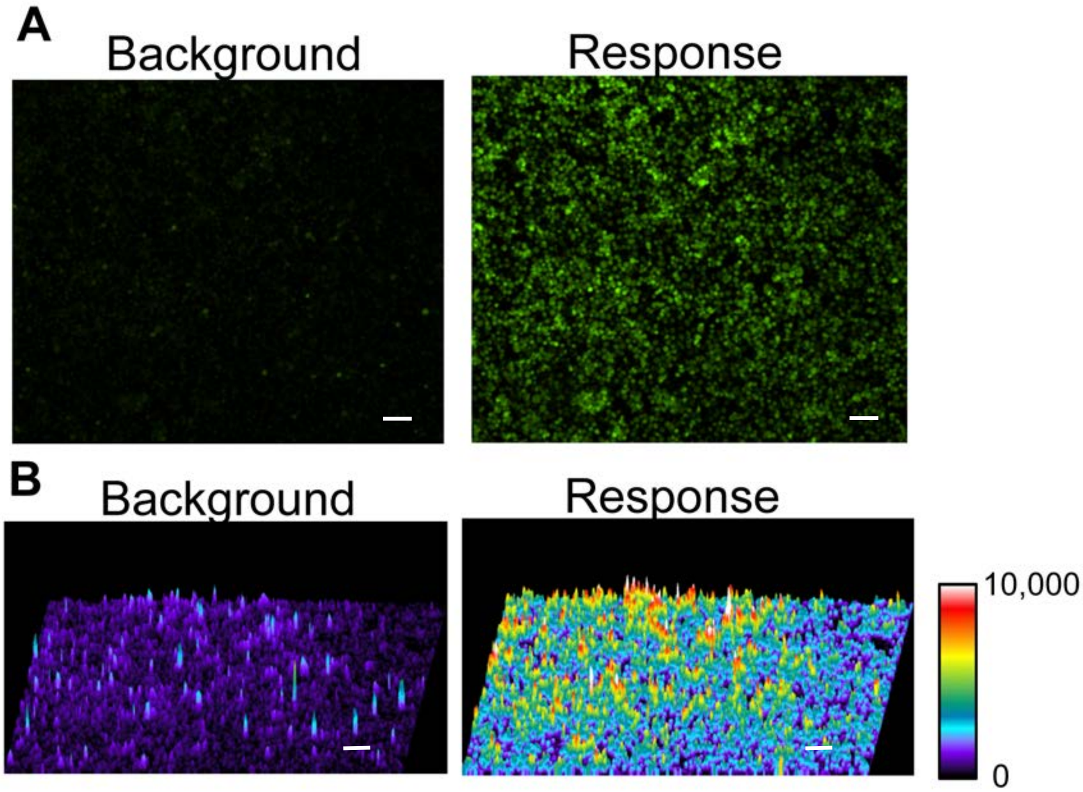

Supplementary Figure 3. Responses of CD8 CTL CER43 to strong agonist GILGFVFTL (GL9) at 10^{-8} M is shown in 2D (A) and 3D (B) plots. Other details are as in Supplemental Fig. 2.

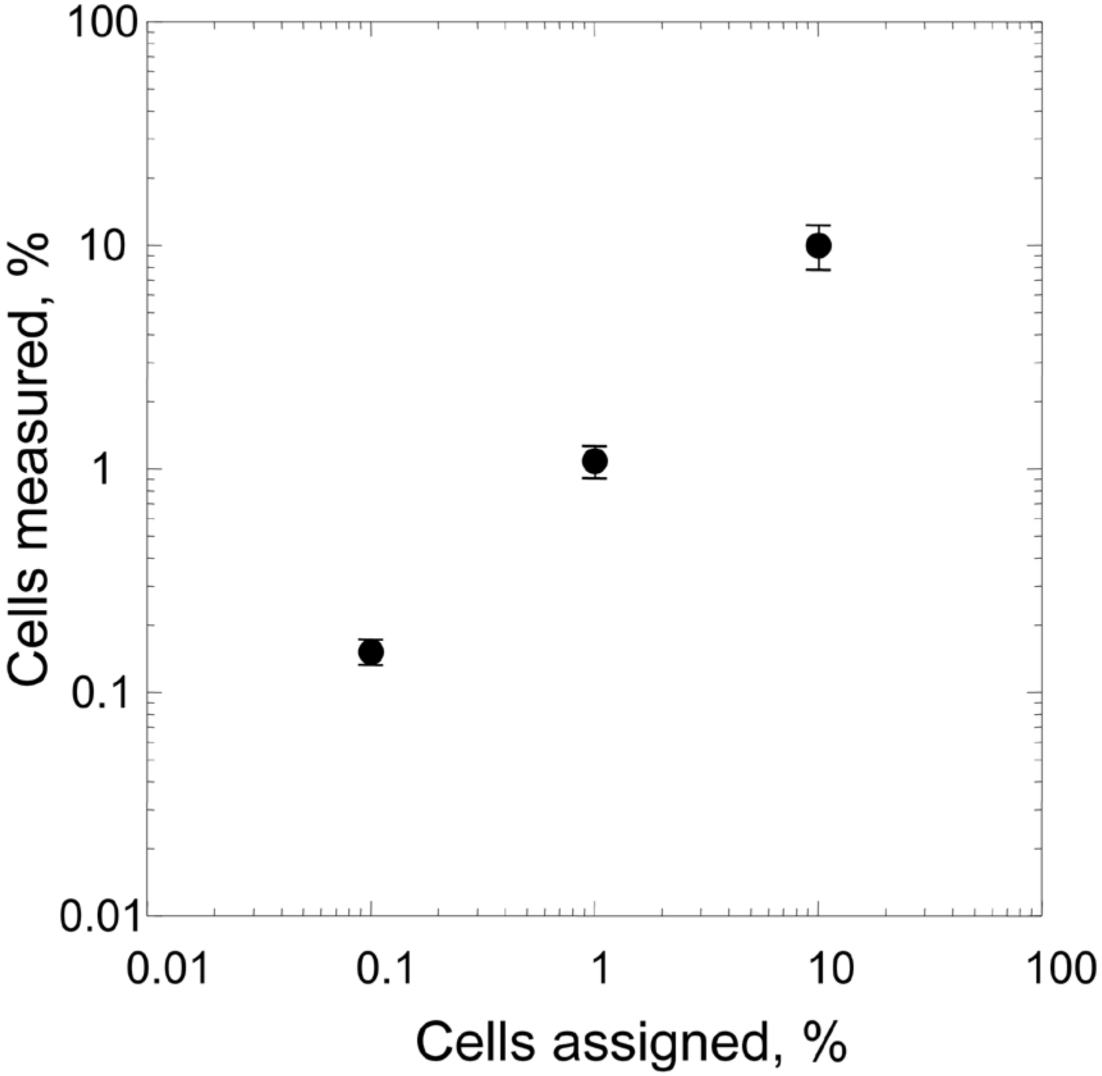

Supplementary Figure 4. Cloned CD8+ CTL 68A62 were serially diluted with irrelevant non-responding T cells and each mixture was immobilized on the glass surface to form a T-cell monolayer as described in Methods. Strong agonist peptide ILKEPVHGL (IL9) was added to the monolayers at 10^{-4} M and the number of responding cells was determined for every dilution. The percentage of responding 68A62 cells was determined in 3 independent experiments and was compared with percentage of assigned 68A62 cells using linear regression function $y = 0.072111 + 0.99718x$; $R^2 = 0.99999$. The difference between percentage of responding cells at lowest dilution and the background was evident from Student's t-test ($p<0.02$).

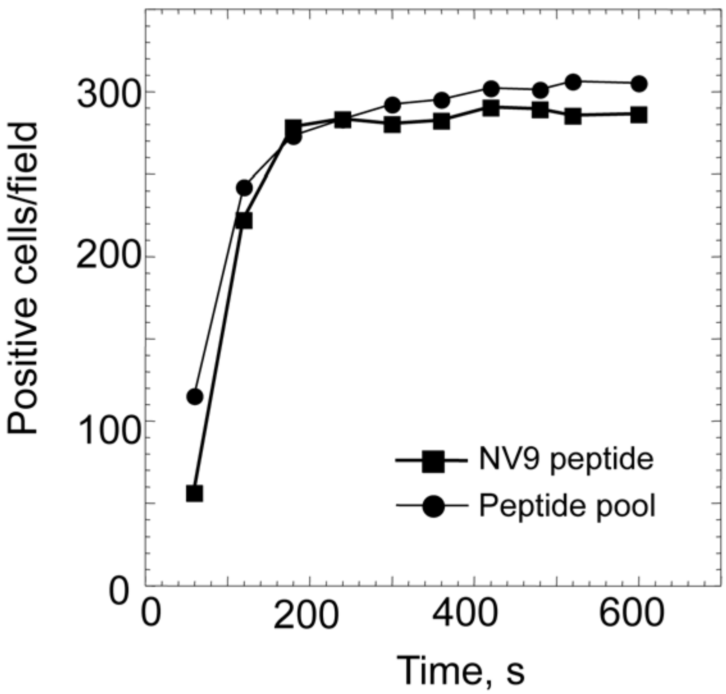

Supplementary Figure 5. Kinetics of calcium response of CMV-specific $CD8^+$ T cells from a healthy donor. CD8+ T cells were purified from PBMC by negative magnetic sorting and purified CD8 T cells were labeled with Fluo-4 and immobilized on a glass surface to form T-cell monolayers. The T cells were then stimulated with individual peptide NLVPMATV (10^{-4} M) or with ProMix CMV peptide pool at $9x10^{-5}$ M containing NLVPMATV peptide at $6x10^{-6}$ M. The dependences of the number of responding cells per field upon time are shown. The data are representative of 2 independent experiments and are based on the analysis of about 5,500 T cells in each case.

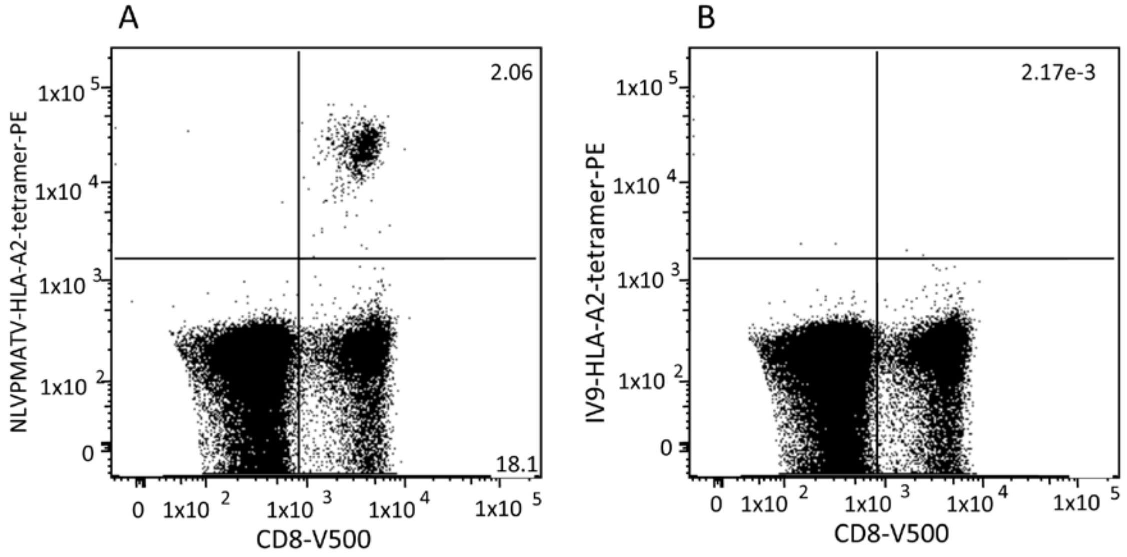

Supplementary Figure 6. Detection of HCMV-specific cells in PBMC of healthy donor by tetramer staining. The PBMC were stained with NLVPMATV-HLA-A2 HCMV tetramer (**A**) and ILKEPVHGV-HLA-A2 HIV tetramer (**B**). The tetramers were prepared as described previously [1-3]. Freshly thawed PBMC were resuspended at $1x10^7$ per mL in DPBS with 1% BSA. 100 µl of PBMC per sample were stained for 30 min at room temperature with HLA-A2-tetramer-PE loaded with HCMV-derived peptide NLVPMATV or HIV-derived peptide ILKEPVHGV (negative control). At the end of the incubation, V500 labeled antibody against CD8 (SK1 clone, BD Horizon) and PerCP-Cy5.5 labeled antibody against CD3 (UCHT1, BD Pharmingen) were added to the incubation mixture for 20 min. Samples were washed and analyzed on BD LSR II Flow cytometer. The gating strategy included FSCxSSC gating followed by $CD3^+$ gating. At least 30,000 cells were collected for each sample. The data were analyzed using FlowJo Software. Percent CD8 T cells and tetramer positive cells among CD3-positive cells are shown.

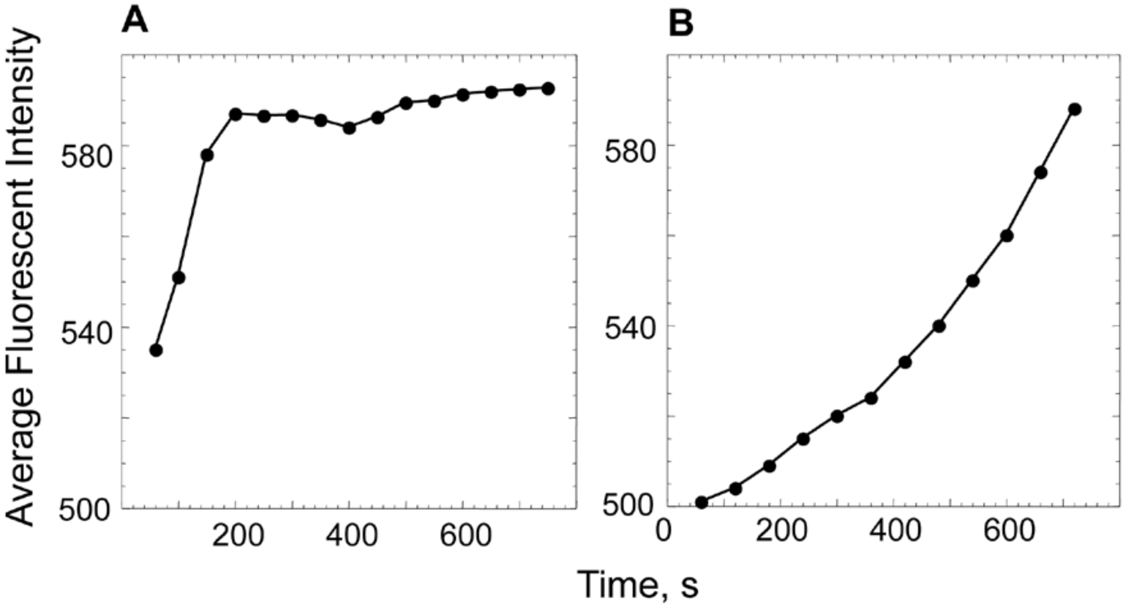

Supplementary Figure 7. Kinetics of calcium response of CMV-specific $CD8^+$ T cells from a healthy donor (**A**) or a patient after marrow transplantation (**B**). CD8+ T cells were purified from PBMC by negative magnetic sorting. Purified CD8 T cells were labeled with Fluo-4 and immobilized on a glass surface to form T-cell monolayers. The T cells from healthy donor were stimulated with CMV-derived peptide NLVPMATV at 10^{-4} M and the T cells from the patient were triggered with ProMix CMV peptide pool at $9x10^{-5}$ M. The dependences of the average fluorescent intensity of analyzed images upon time are shown. The data are representative of 5 (panel **A**) and 2 (panel **B**) independent experiments and are based on the analysis of 4,600 and 4,200 T cells, correspondingly.

Supplementary Tables

Table 1. The number of responding T cells per imaging field

Cells	Field Number	Responding cells per field (≈10^4 CD8 T cells)	Average number of responding cells, mean±SD
CER43:115iX 1:100	1	126	123±7
	2	115	
	3	128	
CER43:115iX 1:2,000	1	5	4.3±1.1*
	2	5	
	3	3	
115iX	1	1	1.7±1.1*
	2	1	
	3	3	

CER-43 is antigen specific (responding) clone;
115iX is unresponding clone. *$p<0.05$

Table 2. Response of healthy donor T cells to HCMV peptide NV9*

	Tetramer NV9-HLA-A2	ELISPOT NV9	CaFlux NV9	CaFlux Peptide mixture****
% $CD3^+CD8^+$ cells	9.95±0.35	2.38**	4.92±1.12***	5.51±0.39
SFS per $2x10^5$ PBMC	NA	494±17	NA	NA

*The data represent pooled average of 2 to 4 independent experiments and are shown as mean±SD

**Numbers indicate the value recalculated as % of $CD8^+$ T cells

***Percent responding T cells was determined as a ratio of the number of responding cells divided by the total cell number in the field

****ProMix CMV peptide pool

Table 3

HCMV peptide pool from ProImmune

Peptide	Sequence	Epitope source	HLA allele restriction
1	VTEHDTLLY	pp50 245-253	A*01:01
2	**YSEHPTFTSQY**	pp65 363-373	A*01:01
3	NLVPMVATV	pp65 495-504	A*02:01
4	VLAELVKQI	IE1 81-89	A*02:01
5	VLEETSVML	IE1 316-324	A*02:01
6	KLGGALQAK	IE1 184-192	A*03:01
7	QYDPVAALF	pp65 341-349	A*24:02
8	VYALPLKML	pp65 113-121	A*24:02
9	RPHERNGFTVL	pp65 265-275	B*07:02
10	TPRVTGGGAM	pp65 417-426	B*07:02
11	QIKVRVDMV	IE1 88-96	B*08:01
12	SDEEEAIVAYTL	IE1 378-389	B18
13	EFFWDANDIY	pp65 512-521	B27
14	IPSINVHHY	pp65 123-131	B*35:01

CMV-derived peptides highlighted in red could be presented by MHC class I proteins HLA-A*01:01, which are expressed on the T cells derived from a patient who underwent bone marrow transplantation.

Supplementary References

1. Anikeeva, N. et al. Distinct molecular mechanisms account for the specificity of two different T-cell receptors. *Biochemistry* 42, 4709-4716 (2003a).
2. Anikeeva, N., Lebedeva, T., Sumaroka, M., Kalams, S.A. & Sykulev, Y. Soluble HIV-specific T-cell receptor: expression, purification and analysis of the specificity. *J. Immunol. Meth.* 277, 75-86 (2003b).
3. Anikeeva, N., Gakamsky, D., Scholler, J. & Sykulev, Y. Evidence that the density of self peptide-MHC ligands regulates T-cell receptor signaling. *PLoS One* 7, e41466 (2012).
